# StrandAdvantage test for early‐line and advanced‐stage treatment decisions in solid tumors

**DOI:** 10.1002/cam4.1037

**Published:** 2017-04-03

**Authors:** Manimala Sen, Shanmukh Katragadda, Aarthi Ravichandran, Gouri Deshpande, Minothi Parulekar, Swetha Nayanala, Vikram Vittal, Weiming Shen, Melanie Phooi Nee Yong, Jemima Jacob, Sravanthi Parchuru, Kalpana Dhanuskodi, Kenneth Eyring, Pooja Agrawal, Smita Agarwal, Ashwini Shanmugam, Satish Gupta, Divya Vishwanath, Kiran Kumari, Arun K. Hariharan, Sai A. Balaji, Qiaoling Liang, Belen Robolledo, Vijayashree Gauribidanur Raghavendrachar, Mohammed Oomer Farooque, Cary J. Buresh, Preveen Ramamoorthy, Urvashi Bahadur, Kalyanasundaram Subramanian, Ramesh Hariharan, Vamsi Veeramachaneni, Satish Sankaran, Vaijayanti Gupta

**Affiliations:** ^1^ From Strand Life Sciences5th Floor, Kirloskar Business ParkBangaloreIndia; ^2^ Mazumdar‐Shaw Center for Translational Research (MSCTR)Mazumdar‐Shaw Medical Foundation^th^A‐Block, 8^th^ Floor #258/A, NHHealth CityBangaloreIndia; ^3^ Strand Life Sciences12635 E. Montview Blvd.Suite 360AuroraColorado80045; ^4^ ProPath LLCDallasTexas

**Keywords:** Clinical utility, immunohistochemistry, next‐generation sequencing, solid tumors, standard‐of‐care therapy

## Abstract

Comprehensive genetic profiling of tumors using next‐generation sequencing (NGS) is gaining acceptance for guiding treatment decisions in cancer care. We designed a cancer profiling test combining both deep sequencing and immunohistochemistry (IHC) of relevant cancer targets to aid therapy choices in both standard‐of‐care (SOC) and advanced‐stage treatments for solid tumors. The SOC report is provided in a short turnaround time for four tumors, namely lung, breast, colon, and melanoma, followed by an investigational report. For other tumor types, an investigational report is provided. The NGS assay reports single‐nucleotide variants (SNVs), copy number variations (CNVs), and translocations in 152 cancer‐related genes. The tissue‐specific IHC tests include routine and less common markers associated with drugs used in SOC settings. We describe the standardization, validation, and clinical utility of the StrandAdvantage test (SA test) using more than 250 solid tumor formalin‐fixed paraffin‐embedded (FFPE) samples and control cell line samples. The NGS test showed high reproducibility and accuracy of >99%. The test provided relevant clinical information for SOC treatment as well as more information related to investigational options and clinical trials for >95% of advanced‐stage patients. In conclusion, the SA test comprising a robust and accurate NGS assay combined with clinically relevant IHC tests can detect somatic changes of clinical significance for strategic cancer management in all the stages.

## Introduction

Genetic profiling of tumors is rapidly gaining acceptance in identifying drug targets for therapy. In recent times, the rate of targeted therapy approvals by the Food and Drug Administration (FDA) has outpaced that of chemotherapy approvals (http://www.cancerprogress.net/cca/how-far-weve-come-decade-review) and is indicative of a paradigm shift in cancer therapeutics. In addition to detecting mutations indicative of chemotherapy response, aggressive phenotype, and poor prognosis, tumor profiling helps identify direct and/or indirect drug targets using a pathway‐based approach. An emerging hypothesis is that cancers coalesce into “common subtypes” based on molecular markers, in addition to classical subtyping by tissue of origin 1. This molecular taxonomy of cancer allows drugs initially approved in one cancer to be expanded for clinical trials and usage in other cancers if the target is similarly activated (off‐label drugs) 2, 3, 4. Identifying key mutations and relating them to treatment options and prognosis is at the core of precision medicine for better disease management.

Tumor heterogeneity and normal cell contamination in tumor samples necessitate the need for a highly sensitive test. Single‐locus molecular tests, like quantitative PCR (qPCR) and droplet digital PCR (ddPCR), have very high sensitivity but are low throughput. Testing multiple genes and loci in a single reaction is only possible by either microarrays or next‐generation sequencing (NGS) 5. While arrays are developed based on known variants, NGS can identify novel variants that potentially affect protein function. The targets of an NGS‐based test 6, 7, 8, 9 could range from mutational hotspots to the entire exome allowing flexibility in the selection of gene targets depending on the clinical utility of the test.

In addition to DNA markers, a significant number of protein markers 10, 11, 12 impact early‐line or standard‐of‐care (SOC) therapy. Immunohistochemistry (IHC) is best suited to detect protein expression levels. An NGS‐based sequencing test that spans multiple relevant coding exons, combined with traditional IHC to evaluate protein expression status, can provide the necessary information to appropriately tailor treatment in all stages of cancer.

In this article, we present the analytical validation and clinical utility of the StrandAdvantage (SA) test that combines NGS‐based sequencing of 152 genes involved in all approved targeted therapies and most drugs in clinical trials, along with IHC to provide a comprehensive molecular view of tumors, thus enabling decision making for both early‐line and late‐stage treatment. Massively parallel sequencing is performed on all exons that carry mutations covered by COSMIC database v.68, including relevant single‐nucleotide variants (SNVs), insertions/deletions (InDels), copy number variants (CNVs), and structural variants (SVs). This is coupled with protein expression data for selected IHC markers, with relevance to tumor type‐specific chemotherapy and targeted therapy. In addition, a PCR‐based microsatellite instability (MSI) test is also included for colon cancer due to its relevance to chemotherapy.

The characteristics of the NGS assay have been validated for analytical performance, and bioinformatics pipelines have been tuned to detect all variant types. A subset of different variant types detected by this assay in 46 clinical samples have been confirmed using range of orthogonal methods like qPCR, FISH, Agena's MassArray, and pyrosequencing. Sensitivity for SNPs/InDels was established across 1982 distinct loci. Specificity was established across 14,836 characterized loci. For CNV and SV, a total of 10 loci were used for sensitivity and specificity calculations as very few control samples (cell lines) with these rare events exist. Thirty‐one copy number calls were validated across 19 cases. A wide variety of validation samples including commercially available reference controls, pools of cell lines, clinical samples, and unaffected tissue samples were used for the analytical validation.

NGS‐based testing of cancer samples poses challenges in variant detection that necessitate extensive validation of the NGS analysis pipeline. Obtaining fresh tissue samples is not practical in clinical settings. Formalin‐fixed paraffin‐embedded (FFPE) samples are routinely available but pose a different set of problems. Improper fixation causes severe damage to the nucleic acids in these samples and results in DNA fragments of shorter length. There are also cross‐linking and deamination effects leading to an increased C > T conversion rate 13. Trending analysis of the percentage of C > T variants in control samples helps in setting thresholds for this conversion rate, thus avoiding reporting of false positives.

The variant data are then interpreted using indigenously developed software (StrandOmics, v.3.1) that not only prioritizes the variants based on therapy options and tumor type, but also integrates data from NGS, IHC, and MSI tests into a consolidated report. The interpretation process involves integration of both the NGS variant information as well as evaluation of protein marker expression status to provide a report with recommendations of therapy options that might work effectively or not; in addition to providing prognosis level information that make certain tumors refractory to therapy. This comprehensive reporting and its clinical utility have not been previously evaluated extensively in literature.

The StrandAdvantage test results are reported in two stages: 1 standard‐of‐care (SOC) and 2 investigational reports, currently for four solid tumor types, namely breast, lung, colon and melanoma. The SOC report is available in 10 days, with mutations affecting genes that are implicated in response to drugs prescribed in treatment guidelines such as National Comprehensive Cancer Network (NCCN) (https://www.nccn.org/professionals/physician_gls/f_guidelines.asp). This is followed by an investigational report within the next 2 weeks, covering all mutations impacting therapy or prognosis, including drugs in appropriate clinical trials. For all other solid tumor types, a single investigational report is issued with a 21‐day turnaround time.

## Methods

### Control samples used for analytical validation of the NGS test

Control samples with known mutations were obtained either as cell lines (HCC1143, HCC2218, HCC827, J.RT3‐T3.5, HCC2228, Jurkat, LC‐2/ad, LoVo, MDA‐MB‐435) from ATCC (Manassas, VA) or isolated genomic DNA (NA12878, NA12879, NA16533, NA18523, NA18912, NA19190, NA20127, NA20813, NA24143, NA24631, HG02322, HG02497, HG02769, HG03052, HG03091, HG03198, HG03575, HG03616) from Coriell Institute for Medical Research (Camden, NJ) and Sigma (St. Louis, MO). Cells were cultured as per the recommended protocol. Quantitative Multiplex Reference Standard (QMRS, Horizon Control HD200), Genome‐in‐a‐Bottle controls (GM24143, GM24385) were purchased from Horizon Discovery Group (Cambridge, UK). FFPE normal tissues (breast, colon, kidney, liver, lung, pancreas, prostate, bladder, lymph node, spleen) were obtained from BioChain Institute, Inc. (Newark, NJ). The details of the samples appear in Table S1A. Raw data (FASTQ) are available on SRA with serial accession numbers from SRR4417035 to SRR4417123 (BioProject accession number: PRJNA344853).

For sensitivity analysis, we used 19 different cell lines pooled to create a range of variant frequencies expected in somatic cancer samples. Cell line pools were created using precharacterized HapMap 1000 Genome samples (pool 1 and pool 2) and ATCC cell lines (pool 3) for SNVs, control cell lines HCC1143, HCC2218, and A549 for CNVs, and HCC2228 and LC‐2/ad diluted with NA12878 for SVs. For specificity, NA12878 and HD200 for SNVs, NA12878 and NA12879 for CNVs, and Biochain samples for SVs were used.

### Clinical samples for validation of the SA test

Clinical samples were obtained from two sources. Forty‐six retrospective samples (Table S2A) across different cancer types were obtained. Ethics committee approval was obtained from participating hospitals and laboratories as well as from Strand's internal ethics committee. Two hundred patient samples were tested at the Strand CLIA laboratory (Denver, CO, USA) from various hospitals and physicians in the United States (Table S2B). All samples had >20% tumor content as estimated by histopathological evaluation. DNA was extracted from five 10‐micron curls from FFPE blocks. The reported variants (SNVs, SVs, and CNVs) for clinical samples are listed in Tables S2A and B.

### Selection of genes for the SA test

Genes involved in cancer therapy or other categories were selected using literature survey. A complete list of the 152 genes assayed by NGS and their functional relevance with respect to therapy and prognosis is listed in Table S3. Genes were grouped into those associated with 1 approved targeted therapies (50.7%), 2 drugs in clinical trials (19.1%), 3 drugs in preclinical development (2%), chemotherapy (16.4%), 4 drug metabolism conferring drug resistance (9.9%), and 5 prognosis (2%). Percentage of genes in each category is mentioned in parenthesis. Selection of IHC markers and their relevance to the cancer tissue is given in Table S4.

### Definition of SOC test

A survey of literature enabled the definition of a subpanel of relevant markers specific to each tumor type. A list of standard FDA‐approved and NCCN guideline recommended therapies currently used for treatment for the specific tumor type was compiled. Evidence from literature associating these markers with these therapies was ordered into the following categories:

1. Substantial clinical evidence in the same tissue type.
2. Substantial evidence in the same as well as a different tissue type.
3. Moderate clinical evidence in the same tissue type supplemented with preclinical evidence in other tissue types.

The appropriate testing method was chosen (NGS for mutations and IHC for proteins) depending on whether the evidence was related to gene mutation or protein expression, respectively. The list of SOC markers for breast, lung, colorectal, and melanoma are listed in Table S4.

### NGS panel design

Hybridization capture probes were designed on the 152 genes to cover all exons (reference genome build: NCBI HG‐19) spanning any reported somatic mutations in the COSMIC database (v.68) with standard 1× tiling using the Agilent SureDesign Software (v.3.0). For tumor suppressor genes all coding exons (RefSeq hg19) were included. Exons were extended to include 10 intronic bases on either side to capture splice site mutations. The fusions in three actionable genes were targeted: *ALK, RET,* and *ROS1*. The assay is designed such that it includes probes that cover all the known breakpoints in these three genes (from COSMIC) irrespective of the fusion partner, novel or known. By this virtue, any partner for these genes fused at the probe‐targeted breakpoint for *ALK, RET,* and *ROS1* can be picked up by the hybridization probes. A schematic to illustrate this design strategy is shown in Figure S1.

For genes with reported copy number variations, multiple regions of variable length interspersed across coding and noncoding regions of the gene were included. The total number of target bases for this multigene panel was ~500 kb.

### DNA isolation and NGS protocols

FFPE DNA from control and clinical samples were extracted using Qiagen AllPrep DNA/RNA mini kit (Qiagen, Germany) as per manufacturer's recommendations. Extracted DNA was quantified using Qubit dsDNA HS assay (Thermo Fisher Scientific, Waltham, MA, USA) and assessed for fragmentation on TapeStation (Agilent Technologies, Santa Clara, CA). The degree of cross‐linking was assessed using qPCR‐based Illumina Infinium assay (Illumina, San Diego, CA). Samples with <2∆Ct were taken forward. FFPE DNA (200–1000 ng) was sheared using Covaris M220 (Covaris, Woburn, MA) for 160 sec at 20% duty factor. The number of PCR cycles to obtain indexed precapture libraries was fixed at eight cycles. Sheared DNA was used for indexed library preparation using SureSelect XT2 kit (Agilent Technologies). Uniquely indexed libraries were pooled to obtain six to eight samples per pool, corresponding to a total of 1500 ng. Targets were pulled down in solution using SureSelect XT2 RNA baits and captured as per manufacturer's recommendation.

The final library was sequenced on MiSeq using 2*151 V3 (Illumina) chemistry. The loading was optimized to get 10–15 million reads per sample. The demultiplexed FASTQ files were used for downstream analysis, quality control, and parameter optimization in the Strand NGS (Strand Life Science, Bangalore, India) software.

### Immunohistochemistry protocols

The protocol for IHC was standardized by ProPath Laboratory (Dallas, TX). A multi‐tissue control block that contained 10–80 pieces of known positive and negative control tissues was used for standardization. Different antigen retrieval methods were tested along with different antibody dilutions to arrive at a standardized protocol. Interpretation of the results being positive or negative for clinical samples, for each stain, was determined as described in Table S5.

The markers included were estrogen receptor (ER), progesterone receptor (PR), HER2, P‐glycoprotein, PTEN, topoisomerase 2A (TOP2A), tubulin B3 chain (TUBB3), thymidylate synthase (TS), ERCC1, topoisomaerase 1 (TOP1), PD‐L1, and TLE3. Sources of the antibodies are listed in Table S6.

FFPE tissues were sectioned at 4 microns and mounted on adhesive slides, along with sample being studied. After drying, the slides were deparaffinized in xylene and rehydrated in graded alcohols to distilled water. Endogenous peroxidase activity was quenched for 10 min at room temperature, using 0.3% H_2_O_2_ with 0.1% sodium azide added. Antigen retrieval was carried out using 1 mmol/L EDTA, pH 8.5 for 30 min for all markers except RRM1. Tris‐base buffer (0.25 mol/L) was used for RRM1. After rinsing the slides in phosphate‐buffered saline (PBS), primary antibody incubation was performed for 50 min at 25°C in an incubation oven, using gentle orbital rotation at 40 rpm. Following another rinse in PBS, incubation with the appropriate antimouse or antirabbit horseradish peroxidase‐conjugated polymer (PowerVision Poly‐HRP anti‐Mouse IgG or anti‐rabbit IgG, Novocastra Reagents, Leica, Buffalo Grove, IL) was performed for 45 min at 25°C, using gentle orbital rotation at 40 rpm. The slides were developed using diaminobenzidine (DAB) (Invitrogen, Carlsbad, CA), enhanced with 0.5% copper sulfate in PBS for 3 min at 25°C, counterstained in hematoxylin, dehydrated in graded alcohols, cleared in xylene, before imaging.

### Microsatellite instability (MSI) test

DNA was extracted from areas of “normal” and “tumor” tissue, and amplified using the MSI Analysis System (Promega Corp., Madison, WI). Amplification products were analyzed by capillary electrophoresis. This test was developed and its performance characteristics determined by ProPath Services, LLP.

### NGS data processing pipeline

**Figure 1:**
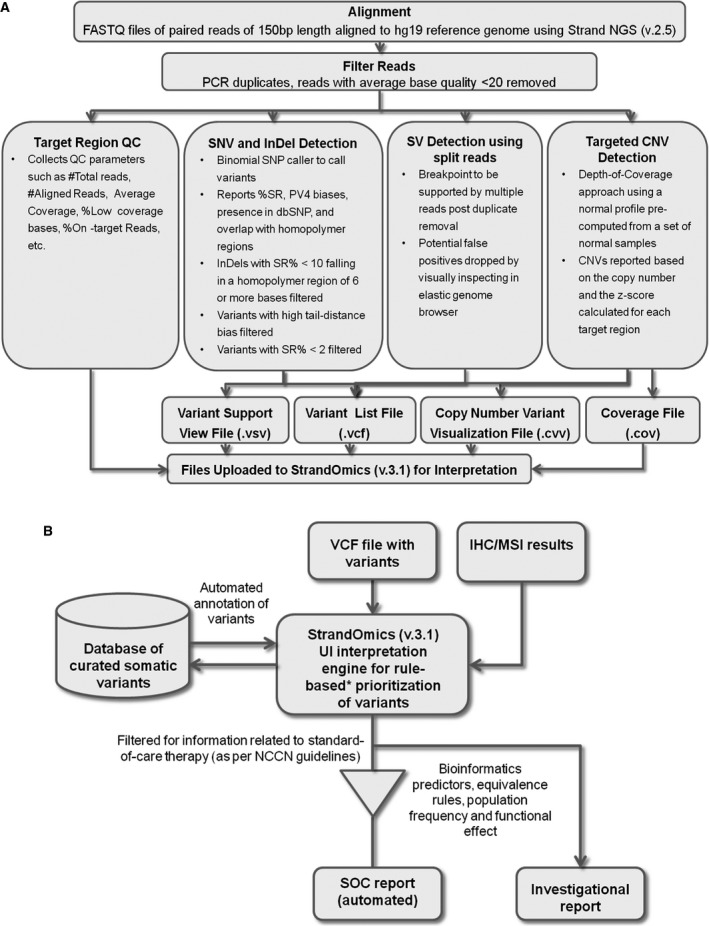
Workflow for data analysis and interpretation pipeline for the SA test. (A) Sequencing run data are demultiplexed using MiSeq reporter software and FASTQ files are uploaded in Strand NGS software for alignment against human HG19 reference genome. Following duplicate removal, QC checks on the target region, variant detection, copy number calls in target genes, and SV detection are performed in the software using an automated analysis pipeline. Four files are generated after processing the data. The VCF file containing information on all classes of variants, and the supporting visualization files (VSV, variant support view; CVV, copy number variant visualization) and the COV coverage files for the target regions are uploaded into StrandOmics (v.3.1) interpretation and reporting software. (B) The four files from Strand NGS software along with any results from IHC, FISH, or MSI tests for SOC cases are uploaded into StrandOmics software. Variants are prioritized based on the rules encoded within the software. The variants present in the patient that can potentially influence the response to a SOC drug are automatically identified within the software. Following manual inspection, an SOC report is generated within 10 working days. The full list of NGS variants is further categorized by likely functional effect (gain of function, loss of function, damaging or likely benign). This is done by comparing the variants against a curated database and by further applying equivalence rules, bioinformatics predictors, and population frequency filters. The most relevant variants are prioritized, manually inspected, and are associated with relevant therapies and drugs in clinical trials within the software. A full investigational report is generated with a turnaround time of 21 working days. IHC, immunohistochemistry; SOC, standard‐of‐care; SV, structural variants.

The NGS data analysis pipeline is shown schematically in Figure 1A. The development of the pipeline is described below.

#### Alignment and read filters

Paired reads of 150 bp length from the sequencer were aligned against the HG19 reference genome using Strand NGS (v.2.5). The alignment procedure first finds locations in the reference genome where a subsequence of the read, called a seed, matches perfectly, using the Burrows–Wheeler transform (BWT) index, and then performs a dynamic programming (DP) procedure at such locations where the exact match is of maximal length to identify read matches with at most 5% mismatches and 45% gaps. Reads that matched the genome at more than five places with alignment score >95% were ignored. If multiple matches with alignment score >95% were found, then it assigned a mapping quality that indicated the number of such matches. If a read pair did not get a pair of matches for the two reads such that the matches have the correct orientation and are within 6 standard deviations of the expected insert length from each other, then such read pairs were subjected to a rescue process to see if they have any matches with correct orientation and expected separation. Finally, reads that were still unaligned are subjected to a split read procedure, where the read was split into two parts, each of which was independently aligned allowing for at most 3% mismatches and 5% gaps. The split itself was determined based on a read prefix or suffix matching substantially, as discovered in the DP procedure above.

Once the reads were aligned, they were filtered to remove PCR duplicates. Read coverage of target regions was then assessed to identify average coverage, off‐target leakage, and the number of bases with inadequate coverage.

#### SNV and InDel calling

For detecting somatic variants, an adaptation of the standard binomial SNV calling method was used, to take the base qualities into account. The binomial SNV calling method, like the Bayesian approach, considers the base taken by each read covering the location, as well as its associated base quality, denoted collectively by D, to decide whether there is a variant at that location. But the binomial caller does this by computing the probability *P* of observing a data *D*’ with the same total number of bases and at least as many variant bases as *D* has, with the assumptions that the location has the reference genotype, and that the variants are due to sequencing error only. Only the variant allele with the highest frequency of occurrence was considered for the computation of the probability *P* and the other variant alleles are ignored. This probability was then converted into a score as −10 log_10_ *P*. If this score is greater than 50, then the location was reported as a variant location.

The standard binomial test for calling SNVs requires one overall error rate and cannot use the qualities of all the individual bases. One way to arrive at the overall error rate from the base qualities in *D* was to compute the error rate corresponding to the lowest base quality from *D*. But this may make the results very sensitive to any noisy outlier bases with very low quality leading to false negatives. In order to avoid this problem, all the bases with qualities less than a threshold *Q*_t_ were ignored, and the binomial test was run with the error rate corresponding to the lowest quality from the remaining bases. To make the test more robust, this process is repeated for multiple values of *Q*_t_ and a variant was reported if it was called at any of the thresholds *Q*_t_. Typically, *Q*_t_ is varied from 20 to 30 and if a variant was called in any of these tests, the method reported the variant and the lowest *Q*_t_ at which the variant was called. In case different variants were called at the same locus in these multiple tests, only the one with the highest score was reported.

For each of the variants called, the method reported some additional attributes such as supporting reads percentage (%SR), strand bias, PV4 biases, presence in dbSNP, overlap with homopolymer regions, etc. The reported variant calls were filtered based on some of these attributes to eliminate potential false positives. Here is a list of some of the filters used:

1. InDels called with a supporting read proportion (%SR) <10% of the total reads were ignored if they lie in a homopolymer region of size 6 or more bases.
2. Variants with very high tail distance bias were dropped. All the variants with *P *< 0.00001 were ignored.
3. In addition, all the variants with %SR <2 are considered as low‐confidence variants and are ignored.

#### CNV calling

Somatic copy number variants were detected using a depth‐of‐coverage (DOC) approach which computes for each genomic region, the ratio of the number of reads in the tumor to that in the matched normal sample. Since sequencing two biological samples—normal and tumor—for each patient is expensive, a precomputed normal profile derived from a collection of normal samples was used. The processing involved computing read counts in the tumor sample in every region of the manifest. Read counts were normalized for each region by taking into account the region coverage, total sample read count, and region size. Normalized read counts were further corrected for GC bias and then compared against the profile created from normal samples. The GC bias correction employed was a mean‐based additive correction method 14. The profile consisted of means and standard deviations of the normalized coverage of a set of normal samples for each of the regions in the manifest. The profile was created in an iterative process in which outlier samples were eliminated at each step till there were none. At every step, mean and standard deviation were computed from the remaining samples, and a *Z* score for each region was computed for every sample that indicated deviation from the mean as a multiple of the standard deviation. A region was marked as deviant for a given sample if it exceeded a specified *Z* score cut‐off. A sample with more than a specified number of deviant regions was considered as an outlier sample and is eliminated for the subsequent iteration.

Once the copy numbers for the individual target regions were computed, the copy number for the entire gene was computed as the average of the copy numbers accorded to the constituent target regions of the gene, weighted by their respective region sizes. Amplification was called if the final copy number for the gene exceeded a certain threshold. To restrict the number of false positives, an additional check required that the number of target regions supporting the amplification call exceeded a sufficiency threshold value as well. Deletion calls followed a similar protocol where a call was made if the final copy number for the gene was below a certain threshold. The thresholds were determined using the profile generated from HapMap 1000 Genome samples HG02497, HG02769, HG03575, HG03616, NA18523, NA19190, HG03052, HG03091, HG03198, and NA20813 which did not have any known CNVs in the panel manifest regions.

For clinical FFPE samples, a profile of 10 normal FFPE samples (Biochain, Newark, CA, USA) was created and the copy number and *Z* score values for individual target regions were computed using the approach mentioned above. Any gene which had a copy number value >2.7 for more than 50% of its target regions was a candidate for amplification, and any gene which had at least 1 target region with a copy number value <1.2 was a candidate for deletions. Further verification of the amplification and deletion calls were made by manually looking at the copy number profiles, taking into account the weighted copy number and finally checking against a profile of samples previously sequenced. If the copy number of the target region lay comfortably outside the interquartile range of previously sequenced samples, the call was considered to be of higher confidence.

#### SV calling

The SV calling method used the split reads generated by the aligner for detecting large‐scale structural variations. A cluster of split reads, with one segment lying in one gene and the other in another gene, with breakpoints for both segments consistent within most reads in the cluster, indicated a possible translocation. Potential false positives such as the case where the breakpoints were not consistent across reads, etc., were dropped by visually inspecting them in the Strand NGS Elastic Genome Browser, which allows displaying different genomic regions at different zoom levels simultaneously.

### Interpretation pipeline development

The NGS data interpretation pipeline is shown schematically in Figure 1B. The development of the interpretation software and rules used for reporting are described in the subsequent sections.

#### StrandOmics interpretation software development

StrandOmics (https://clinical.strandomics.com) is a clinical genomics interpretation and reporting platform from Strand Life Sciences. FASTQ files were aligned and analyzed using StrandNGS and variant prioritization and interpretation was done using StrandOmics (v.3.1). The StrandOmics Variant Annotation engine includes algorithms to identify variant impact from both public content (HGMD, ClinVar, OMIM, HPO, dbSNP, 1000 Genomes, Exome Variant Server, and COSMIC, and bioinformatics prediction tools such as SIFT, LRT, PolyPhen HVAR/HDIV, Mutation Taster, FATHMM) and proprietary content on genes, diseases, and therapeutic impact of somatic variants.

The proprietary content was developed using its text mining technology which is integrated with Strand's proprietary data mining platform. The text mining engine can extract key concepts, sentiments, and relationships from textual or “unstructured” data and convert them to a structured format that can be used to create predictive models. In the first pass, PubMed abstracts were processed using this tool to find mutations in key genes. All extracted information was then manually curated for validation. The “interpretation interface” in StrandOmics allows quick filtering and evaluation of variants along with capture of justification for inclusion/exclusion. The “reporting interface” in StrandOmics enables identified variants to be carried into template‐driven reports efficiently.

#### Reporting workflow

A VCF file containing SNVs, small InDels, CNVs, and SVs is uploaded into StrandOmics along with the results of any IHC, FISH, or MSI tests for SOC cases. Based on the SOC rules (see Definition of SOC test section) which are encoded within StrandOmics software, the variants present in the tumor that can potentially influence the response to a SOC drug are prioritized. This workflow is shown in Figure 1B and explained in the section earlier. Once these variants are verified, the software generates an SOC report with a single click. The full list of NGS variants is further made available in the investigational section. Here, the variants are categorized and prioritized based on their likely functional effect (gain of function [GOF], loss of function [LOF], presence in COSMIC database, and predicted effect on protein structure or function such as damaging or likely benign). This is done by comparing the variants against a curated database of variants and further applying equivalence rules in the software, using bioinformatics predictors and population frequency filters. Once the relevant somatic variants are selected, the interpreters can further use the tool to identify potential targeted therapies and associated clinical trials to create the investigational section of the report. Therapeutic recommendations will include drugs which are FDA approved or in the list of NCI clinical trials. Drugs in preclinical stages of development are not included in the report.

## Results

### Performance of the SA (NGS) test on MiSeq

The NGS assay was standardized using DNA from cell lines, simulated FFPE QMRS controls, and FFPE clinical samples. Sequencing data were demultiplexed using the MiSeq reporter software (v.2.5.1), aligned and analyzed on Strand NGS for total, average, and low coverage across target regions in each sample. The mean coverage was higher than 500× across all sample types at 6–8 plex on the MiSeq. All 152 genes showed an average coverage of >250X (*TYMP* at 282× and *CDKN1B* at 1056X; see Table S1C) across the samples used in analytical validation. Despite greater variability in coverage distribution, all FFPE clinical samples had <5% of the target bases covered below 100× and showed comparable performance to cell lines or control FFPE samples (Tables 1 and S1B), thus qualifying the SA test for clinical FFPE samples. All genes and samples in the analytical validation show sufficient coverage to qualify this assay for clinical reporting.

**Table 1:** Coverage statistics for different sample classes

Sample type	Total reads^a^	%Duplicates	%On target reads^a^	Average coverage^a^	%Bases (>100X)	%Bases (>200X)	%Bases (Q ≥ 30)
Cell line	49,161,307	13.5	54.8	781.0	96.3	94.3	94.8
FFPE (nontumor) controls^b^	45,178,634	18.6	49.5	878.6	99.4	95.7	94.3
FFPE (tumor) controls	45,066,636	19.3	46.9	980.9	98.9	92.2	93.9
FFPE Clinical samples^c^	44,356,055	21.9	51.0	840.6	98.7	94.0	94.2

Results are averaged across all samples for each sample class.

a Results are reported after filtering out PCR duplicate reads (see Methods).

b Includes genome in a bottle and normal tissue samples from Biochain.

c Includes QMRS sample containing cancer hotspot mutations from various cell lines.

### Detection of SNVs/InDels

The limit of detection (LOD) of the SNV/InDel variant calls is a combined function of coverage and variant allele frequency. *In silico* downsampling experiments were performed to determine the LOD of the variant calling algorithm. The HapMap sample (NA20813) from the 1000 Genomes Project and the GM24385 FFPE sample were used. Random subsets of reads were chosen to create various derivative samples differing in coverage levels across more than 100 loci. This was done independently for reads carrying the variant allele and reads carrying the reference allele. Different subset sizes were chosen for the two sets of reads so as to reflect different variant allele frequencies. For each derivative sample, variant calls were performed as above, and sensitivity was ascertained as a combined function (probability of detection) of coverage and variant allele frequency or %SR. To test the SNV detection sensitivity of the NGS assay end to end, pools of diluted cell line samples (Table S8A) with precharacterized variant information were crafted. Three separate pools were created to cover a wide range of allele frequencies and InDel lengths. Pools 1 and 2 constituted HapMap 1000 Genome samples, while pool 3 had ATCC cell lines. Variant information for HapMap 1000 Genome samples was obtained from 1000 Genomes database, while variants reported for ATCC cell lines were obtained from the ATCC datasheets.

For InDels, NA20813, NA19190, HG03616, and HG0357 were examined across 91 loci to obtain sufficient number of data points. Data suggest that variants could be called at 4% supporting reads (%SR) with 100% probability at a minimum coverage of 400× for SNVs and 500× for InDels. At the set threshold of 150× minimum coverage, all SNVs and InDels with 7%SR could be called with 100% probability (Table S7).

**Figure 2:**
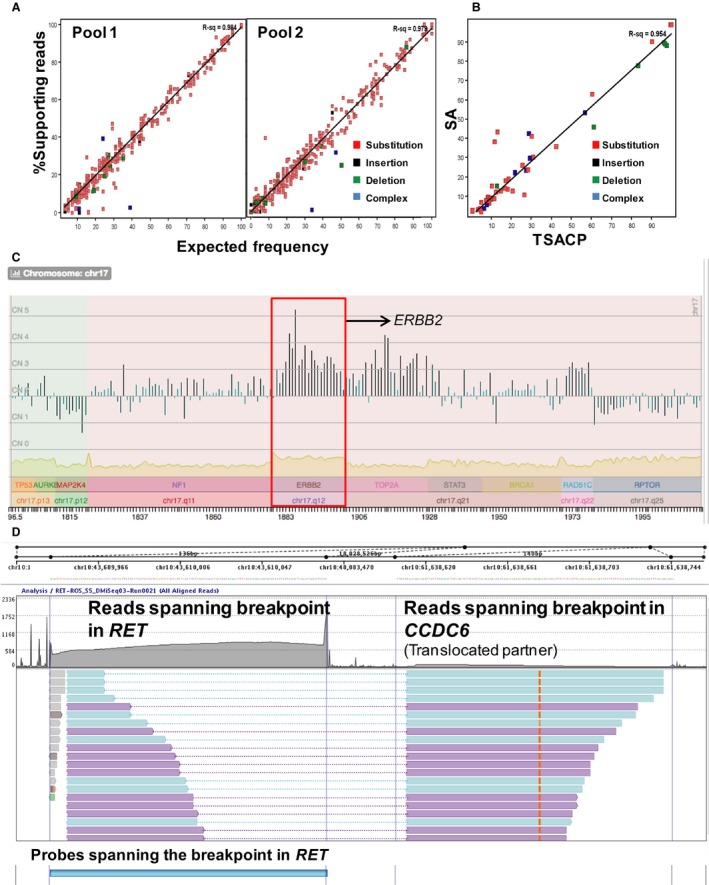
Analytical performance of SA test. (A) SNV concordance between observed versus expected variant frequencies for pools crafted by mixing the constituent samples in different proportions for sensitivity analysis. The different types of variants considered in the analysis are substitutions (red), insertions (black), deletions (green), and complex variants (blue). The *R* ^2^ values for the expected versus the observed frequencies are shown for each pool. (B) Concordance between SNV/InDel frequencies measured by the SA test and the TSACP (Illumina). The scatter plot shows concordance between %SR for SNVs and InDels detected by the SA test and the amplicon‐based TSACP NGS panel for one representative sample. The data points analyzed were restricted to the overlapping regions on both panels. The different classes of variants follow the same representation as in Figure 1A. (C) Copy number variation detection of *ERBB2* in a misclassified sample. The *ERBB2* amplification was detected unambiguously by NGS with a weighted copy number (CN) of 3.2 with 20 of the 23 regions in the *ERBB2* gene having a CN >2.8 with *Z* score >3, thus indicating a significant CNV in the gene in a breast cancer sample. Initially misclassified as *ERBB2* negative by IHC, the test was repeated following the detection of the amplification by the NGS assay and subsequently confirmed by FISH in a repeat test. The *ERBB2* amplification is highlighted in red. (D) Split reads indicating breakpoints in the *CCDC6‐RET* translocation. Probes were designed in very close proximity to known breakpoints in *RET* gene. All fusion molecules having *RET* as one of the partners will be captured by target enrichment using this strategy. In this sample, the split reads that aligned partially on *RET‐CCDC6* and on *CCDC6* each, thereby identifying the chimeric molecule. IHC, immunohistochemistry; SNV, single‐nucleotide variants.

To determine sensitivity, three cell line pools were created (Table S8) using 19 different characterized samples. The SNV/InDel concordance between observed and expected frequencies exceeded the *R* ^2^ value of 0.98, for pools 1 and 2 (Fig. 2A). In pool 3 with only 14 data points, all but one deletion variant was accurately detected. The concordance between replicate experiments was >99%. The results suggest 99% sensitivity for allele frequencies >5% (Tables 2 [SNV/InDel sensitivity] and S9). It is to be noted that the sensitivity of InDels alone is 91.4% because the numbers are low. By combining insertions and deletions we tested 35 loci, of which 32 were detected. One was missed due to an alternate representation, while the other could not be detected due to poor coverage. The longest deletion detected was 35 bp and the longest insertion detected was 39 bp.

**Table 2:** Analytical performance of the SA test

Sensitivity		
SNV/InDel	No. of variants tested	1982
	No. of variants detected	1959
	Sensitivity	>99%
CNV	No. of loci tested	8
	No. of loci detected	7
SV	No. of translocations tested	2
	No. of translocations detected	2
Specificity^a^		
SNV/InDel	No. of bases tested (true negatives)	14,836
	No. of bases confirmed	14,830
	Specificity	>99%
Accuracy^b^		
SNV/InDel	No. of variants tested	31
	No. of variants detected	31
CNV	No. of loci tested	31
	No. of loci detected	27
SV	No. of translocations tested	11
	No. of translocations detected	8
Reproducibility^c^		
SNV/InDel	Intrarun	0.979
	Inter‐run	0.992
	Interoperator	0.976
	Interkit	0.980
	Intermachine	0.977
CNV	Inter‐run and intermachine	0.973

a For CNVs and SVs, samples with no known variants in each class were tested and found to be negative.

b Tested on clinical FFPE samples using pyrosequencing and MassArray for SNVs, qPCR for CNVs, and FISH and Sanger sequencing for SVs.

c Computed *R* ^2^ values for SNV/InDel concordance. SNV, single‐nucleotide variants; SV, structural variants.

SNP detection was performed for control QMRS FFPE sample, with known variants in 24 cancer‐related “hotspot” loci with SR% from 1% to 33%. All variants were detected and matched the expected frequencies well barring two loci where the observed frequency was higher than expected (Table S9B).

The accuracy of SNV calls was established using alternative methods like pyrosequencing, MassArray, or NGS amplicon sequencing. Twenty‐one clinical samples evaluated on the NGS panel were cross verified for 12 clinically relevant loci across six genes (*BRAF*,* EGFR*,* KRAS*,* MET*,* PIK3CA*, and *TP53* ) using pyrosequencing or MassArray. All variants reported by NGS were detected by the second method (Tables 2 [SNV accuracy] and S10) confirming high accuracy of the variant calls. To extend this analysis, the SR% of the variants was compared between the SA test and results from Illumina's 48‐gene TruSeq Amplicon Cancer Panel (TSACP) for five clinical samples. A high concordance, with an *R* ^2^ value in the range of 0.91–0.96 (Fig. 2B), was observed in all cases (Table S10B).

To check for specificity of detection of SNVs and InDels, true negative calls (wild‐type base calls and false positive calls) were analyzed across 12,785 bases in NA12878, and 2051 bases in QMRS FFPE sample. The specificity was determined to be >99% (Tables 2 [SNV specificity] and S11).

### Detection of CNVs

Copy number variations (CNVs) are large amplifications or deletions >1‐kb long. The sensitivity of CNV detection was validated using three precharacterized cell line samples from ATCC, namely A549 15, HCC1143, and HCC2218 16 (Tables 2 [CNV sensitivity] and S12). Amplifications were unambiguously called out for ERBB2 in HCC2218 and all loci in HCC1143, except the SRC locus. The known deletions in HCC1143 (*PBRM1*) and A549 (*CDKN2A*) were called out accurately. The LOD for amplification was found to be 2.7‐fold, while that for deletion was 1.2‐fold. Coriell samples (NA12878 and NA12879) with no known CNV changes in the target regions of the SA test panel did not show any copy number calls indicating high specificity of the CNV algorithm.

To determine accuracy of CNV calls, 31 CNV calls including two copy neutral calls, spanning 15 genes were confirmed using qPCR (Tables 2 [CNV accuracy] and S13). Four exceptions were observed. One borderline *TP53* copy neutral confirmed as a deletion, while a borderline *ERCC1* amplification and amplifications in *MET* and *AKT1* in the same sample did not validate. The qPCR validation results justified the thresholds set for amplification (>2.7‐fold) and homozygous deletions (<1.2‐fold). Concordance between copy number calls by NGS and protein expression status by IHC was analyzed for a couple of markers (ERBB2/HER2 and PTEN) that were tested by both techniques in the SA test. Of the 10 cases reporting either *ERBB2/HER2* amplification or *PTEN* deletions, IHC confirmed 7 *ERBB2/HER2* and 2 *PTEN* CNVs unequivocally. In the tenth sample, IHC reported equivocal expression of ERBB2/HER2, while NGS showed clear amplification (Fig. 2C). The amplification was further confirmed in the sample using FISH upon retesting. Thus, the SA assay was shown to have high sensitivity and specificity with respect to amplifications (≥threefold), and homozygous deletions.

### Detection of SVs

**Figure 3:**
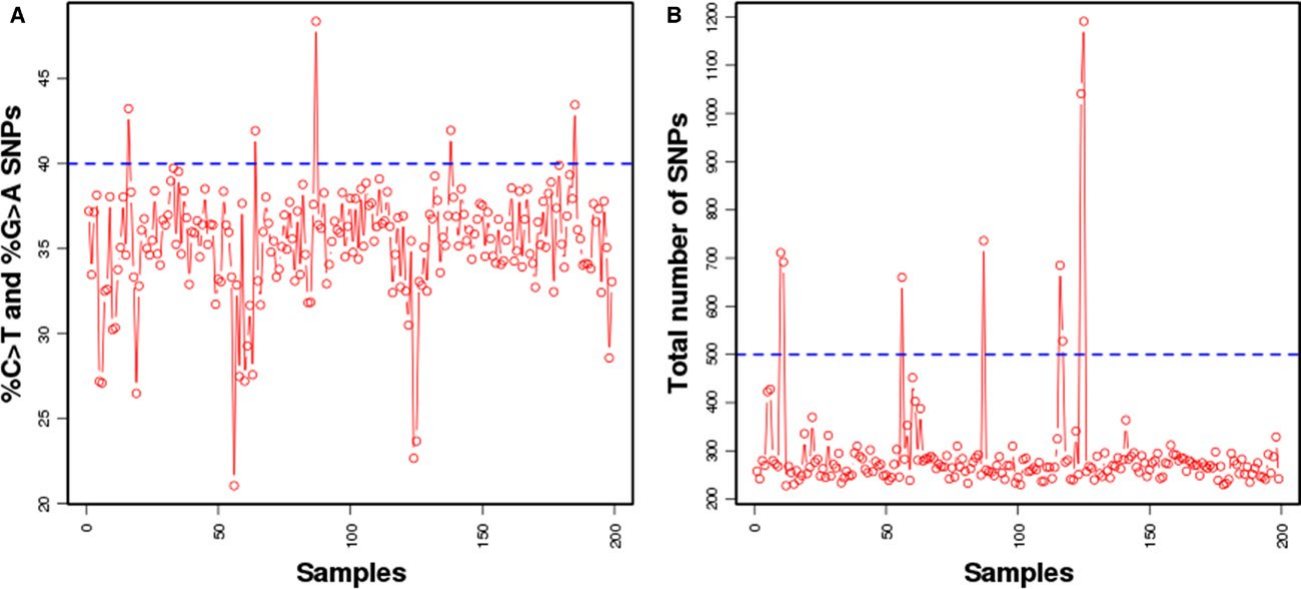
Line plot of (A) percentage C > T conversions and (B) number of variants detected at ≥5% across clinical samples. (A) Percentage of C > T variants is shown as a data point for each sample for SNPs ≥ 5%SR. The threshold is set 40% (blue dotted line) based on cell lines and control FFPE samples. (B) Number of SNPs identified at ≥5%SR for each sample is represented as a data point. The threshold is set at 3 standard deviations of the mean as calculated from cell lines and control FFPE samples as indicated by the blue dotted line.

The sensitivity of SVs (translocations) was established using two cell lines, LC‐2/ad (*RET‐CCDC6*) 17 HCC2228 (*EML4‐ALK*) 18 at 100%, 70%, 50%, and 30% frequencies. Both fusions were detected at all dilutions, with supporting split read numbers inversely proportional to dilution (Tables 2 [translocation sensitivity] and S14). Split reads were confirmed by manual inspection on the Elastic Genome Browser as shown for a sample with *CCDC6‐RET* translocation in Figure 3D.

For accuracy, fusions detected in clinical samples were confirmed using either FISH or Sanger sequencing. Both known and novel translocations were detected, thus validating the probe design strategy. All translocations that could be confirmed had at least six supporting split reads and were confirmed unambiguously at ≥8 (Table S15). The cut‐offs were set accordingly at eight supporting split reads with a second method confirmation recommended for novel translocations. Ten FFPE samples from different tissues (Biochain) were tested with no evidence of split reads supporting the targeted translocations in any of these samples, thus confirming the high specificity of the test.

### Robustness of the SA (NGS) test

To assess the robustness of the SA test, the %SR for variants in QMRS samples were compared in inter‐run, intrarun, interoperator, interkit, and intermachine settings. Metrics of sequencing quality (%base quality ≥30, %on target reads, %duplicate reads, and %coverage <100X) were highly consistent across all comparisons (Table S1B). The *R* ^2^ values for SNV concordance in all comparisons were >0.976 across runs (Table 2 [reproducibility]). This established high reproducibility and robustness of the SA test. For CNV reproducibility, since there is a paucity of positive samples, we generated synthetic samples that represent 20%, 30%, 40%, 50%, 75%, and 100% of tumor cell lines diluted with wild‐type DNA (Table S12). Reproducibility of CNV calls was compared across five positive variants with each of the six dilutions, thereby comparing reproducibility across 30 different data points. As we had only a few positive samples with confirmed SVs, reporting an *R* ^2^ value based on such few data points would be meaningless.

### Detection of sample fixation artifacts

Improper fixation protocols of tumor tissues in formalin leads to deamination of cytosine which contributes to background noise in SNV calling during DNA sequencing. Deaminated cytosines are subsequently read as thymidine during the sequencing steps making it difficult to differentiate from true C>T variants. Both in cell line‐derived DNA and high‐quality FFPE control samples (Genome‐in‐a‐Bottle), percentage of C>T variants was between 35% and 40%, while the overall reported SNVs at ≥5% frequency was ~330. In order to identify compromised samples, the cut‐off for percentage of C>T variants and SNVs at ≥5% frequency observed in clinical FFPE samples was established. It was observed that majority of the clinical samples clustered around 30–40% C>T variants (Fig. 3A). The number of variants with frequency ≥5% was between 250 and 300 for a majority of the clinical samples (Fig. 3B). Since in control FFPE samples the percent C>T conversions never exceeded 45% even with 3 standard deviations, a conservative cut‐off of 45% of C>T conversions was established for qualifying clinical samples for further analysis. The same was set to 500 for the overall number of variant calls.

### Analysis of clinical samples

**Figure 4:**
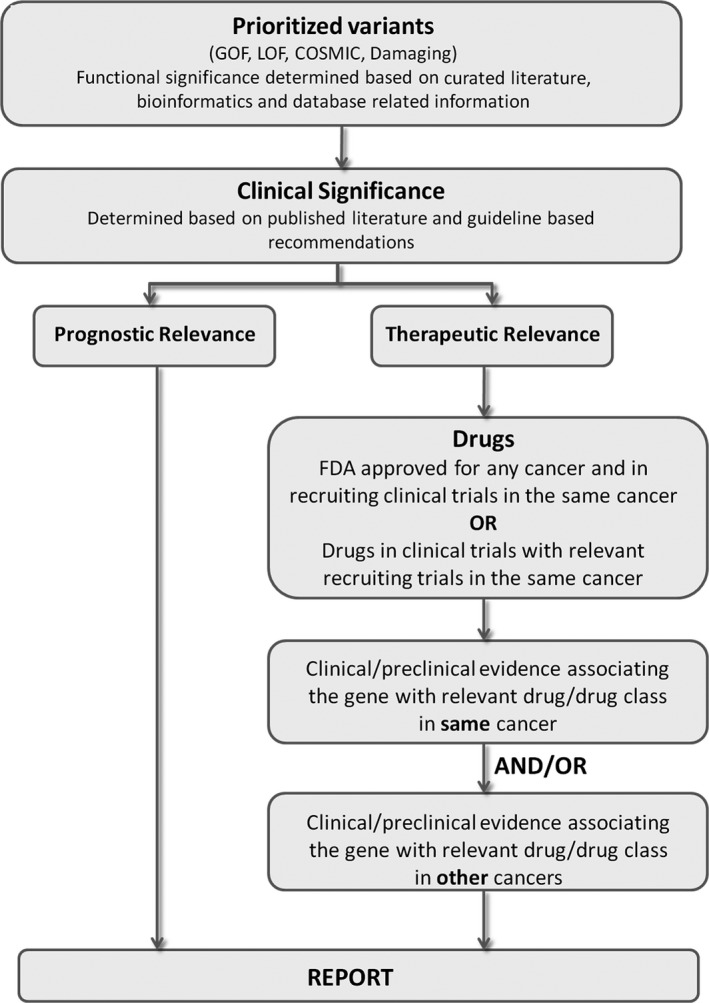
Reporting workflow for somatic variants. All variants outputs from the NGS analysis (.vcf, .cvv) along with their respective visualization files and coverage are uploaded into StrandOmics v.3.1 software. The variants are functionally classified and prioritized with respect to therapeutic or prognostic relevance based on strength of clinical evidence, National Comprehensive Cancer Network guidelines, FDA‐approved therapies, drug repurposing, clinical trials, preclinical testing, etc. The final two‐tier report, standard‐of‐care and investigational, is automatically generated from the software interface.

The SA test was performed on 200 samples at the Strand CLIA laboratory, over a duration of 9 months. Interpretation for these samples was performed as described in the Methods section. A workflow for interpretation of the variants is shown in Figure 4. Briefly, the variants were prioritized for gain or loss of function, damaging effects using appropriate bioinformatics predictors, and presence in COSMIC and other databases. Bioinformatics filters were applied for quality checks on the variants. Next, they were analyzed for clinical significance based on NCCN and FDA guidelines, and published literature for the particular cancer type and an SOC report is generated. Additional therapeutic relevance was determined using data obtained from clinical trials in the same or other tissues and prognostic markers were also shortlisted to generate the final investigation report.

**Figure 5:**
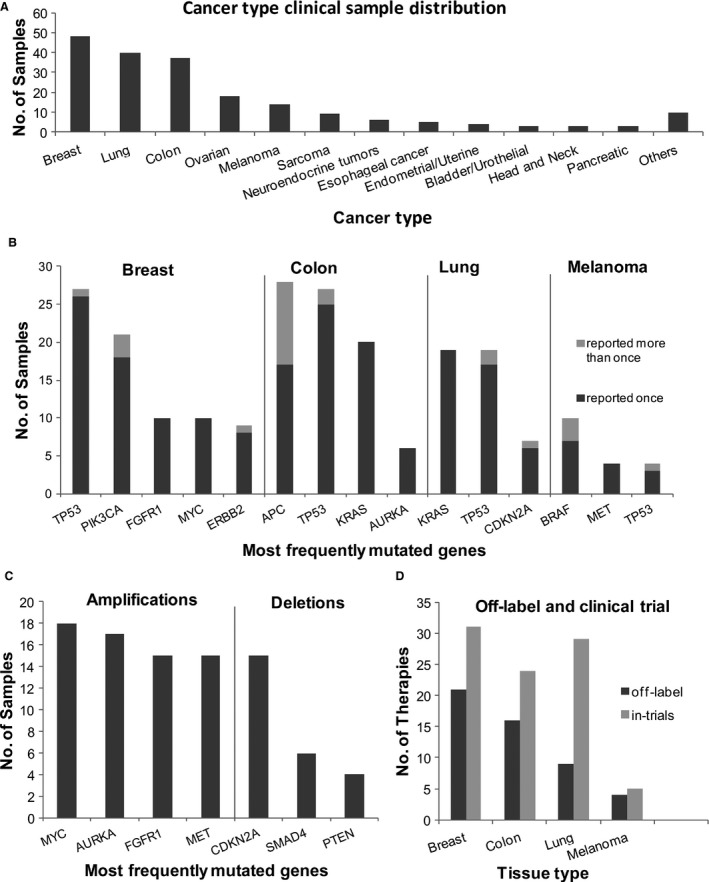
Clinical performance of the SA test. (A) Distribution of tested clinical samples by cancer type. The histogram depicts the distribution of samples processed in the CLIA laboratory by tissue type. Majority of the samples were breast, colon, lung, and melanoma. Next‐generation sequencing was combined with IHC tests for reporting on the standard‐of‐care drugs as well as off‐label drugs and those in trials. The remaining 30% samples were distributed across various tissues. (B) Genes with frequent actionable mutations in breast, colorectal, lung cancers, and melanoma. The histogram shows the number of cases for genes where actionable mutations are reported. Each bar is further classified into those with a single mutation (dark gray) or ≥2 mutations (light gray). Data are provided in Table S18. (C) Frequency of amplified and deleted genes in the clinical samples. The most frequently reported genes with copy number changes (amplifications and deletions) are reported across all cancer samples. (D) Number of off‐label and clinical trial recommendations. Bar graph showing the percentage of samples in breast, lung, colon, and melanoma cancers where off‐label recommendation (approved for a different cancer tissue, indicated in dark gray) of a drug approved or in clinical trial was possible (indicated in light gray). Data are shown for a subset of the total samples processed in the CLIA lab. IHC, immunohistochemistry.

In 193 (96.5%) cases, clinically meaningful information that helped in therapy decisions was reported (Table 2B). Of the samples, 80% were predominantly from five major tissues: breast, colorectal, lung, ovary, and melanoma, while the rest were distributed across other cancers (Fig. 5A). The comprehensive test (NGS and IHC) for the SOC markers was available for breast, lung, colorectal, and melanoma, which comprised 70% of the samples (Table S4), while the NGS assay alone was performed for the remaining 30%.

### Variants detected in clinical samples

*TP53* was the most frequently reported gene across cancer types showing clinically informative mutations. Mutations in *TP53* were reported in 56%, 51%, and 68% of cases in breast, lung, and colorectal cancers, respectively. Equally prominent were genes *PIK3CA* (44%) in breast, *KRAS* (51%) in lung, and *APC* (70%) in colorectal cancers. In melanoma, *BRAF* (71%) followed by *CDKN2A* (21%) were reported with the highest number of mutations (Fig. 5B). The salient observations on clinically relevant SNV/InDels are summarized below for the major cancer tissues:

1. Most cases reported a single clinically relevant variant in a gene. Multiple hits in the same gene were observed for colorectal cancer cases (34%), particularly in *APC* gene (Fig. 5B).
2. In the breast cancer cases, majority of the *PIK3CA* mutations involved histidine at 1047 (47%) converted to either arginine or leucine. This was followed by the Glu545Lys variant (32%) in the same gene (Table S2B).
3. In melanoma, seven of the nine mutations in *BRAF* occurred at codon 600 with, a co‐occurrence of *BRAF* amplification observed in three samples. *BRAF* amplification is a likely mechanism of acquired resistance to *BRAF* inhibitor therapy 19 (Table S2B).
4. Interestingly, in lung cancer cases, *EGFR* exon 19 InDels, the most commonly occurring *EGFR* mutation in lung cancers, was only observed in 5.4% of the cases. Instead, *KRAS* was found to be mutated in 51.4% of the cases. This could be explained by *EGFR* and *KRAS* mutations being mutually exclusive with *KRAS* mutations occurring in western populations more frequently and *EGFR* mutations being more frequent in Asian populations 20.

A total of 114 unique samples reported 241 clinically relevant CNV events (193 amplifications and 48 deletions). These were reported across 47 genes (31 genes with amplifications and 16 genes with deletions), across all cancer samples (Table S2B). Breast cancer samples reported the maximum number of cases with copy number variations (34 of the 48 samples) which accounted for >50% of all reported variants in this cancer type. In contrast, colorectal cancer samples showed significant paucity of amplifications and deletions (14 of the 40 samples). The most frequently amplified and deleted genes are shown in Figure 5C. Across all cancers, the highest number of amplifications were reported in *MYC* (18 cases), followed by *AURKA* 17, *FGFR1* 15, and *MET* 15. More than half the cases reporting *MYC* amplification were from breast cancer (10 of the 18), where it has been reported to cause a highly aggressive form of the disease and has poor prognosis 21. Non–small cell lung carcinoma (NSCLC) cases reported the next highest number of *MYC* amplifications where it has been previously related to metastasis 22. *AURKA* had the second highest number of reported amplifications in clinical samples with most of the cases being either breast or colorectal cancer cases (5 of 17 and 6 of 17, respectively). *AURKA* amplification is positively associated with chromosomal instability (CIN) and poor prognosis in both breast and colorectal cancers 23, 24, 25, 26. The next highest amplifications were reported in *FGFR1* gene and found predominantly in breast cancer cases. This gene has known associations with invasive breast cancer and correlates with poor disease‐free survival 27.

A total of 12 different genes were reported to contain large deletions of clinical relevance. The most frequently deleted gene reported in samples was *CDKN2A* followed by *SMAD4*, and *PTEN*, all of which occurred across a wide variety of tissue types. Translocation were reported in two samples, one was in *ALK* in a lung cancer sample and the other in *RET* in a thyroid cancer case (Table S2B).

### Comparison of clinical utility of the SA (NGS) versus hotspot panels

A vast majority of NGS panels in the market analyze the most frequently mutated genes with clinical relevance. Commercial panels from Illumina (48‐gene TSACP), Agilent (48‐gene Haloplex), and Thermo Fisher Scientific (46‐gene Ion Ampliseq) have >95% overlap in the genes and all of them employ PCR enrichment of mutational hotspot loci. To evaluate additional mutations reported on SA NGS panel vis‐à‐vis the “hotspot” panels, the 48‐gene target amplicon regions from the TSACP panel (Illumina) were overlaid with the target regions of the SA panel. Assuming all target amplicons on the TSACP can amplify at 100% efficiency, all the mutations reported by the SA test in the overlapping regions of the two panels as well as those which are exclusive to the SA test were examined for clinical relevance. The results are summarized below and in Table 3.

1. Additional mutations (SNV/InDels) across 15 genes were reported in 67 distinct cases by the SA test.
2. Eleven distinct additional variants impacting 5‐fluorouracil (5‐FU)‐related therapy decisions were reported in *APC* gene by the SA test, all in colorectal cancer cases 28, 29, 30.
3. Fourteen additional variants across several cancers were reported by the SA test in *TP53* gene, while *CDKN2A* had six (Table S16).
4. None of 150 CNV events in 77 samples or the translocations involving the *ALK*,* ROS1* and *RET* genes reported in three samples would be detected in the hotspot panel. In 12 cases, these were the only reported variants which would be a negative report if run on the TSACP panel.
5. While most samples reported a mutation that would have been detected by the hotspot panel (104 of 134), the SA test supplemented clinically relevant information with additional variants (including CNVs and SVs) in a majority of samples tested (98 of 134). It reported clinically relevant results in 30 samples which would otherwise been negative on TSACP.

**Table 3:** Comparison of clinical utility of SA to TSACP hotspot panel

Parameters	No. of cases
No. of cases used in comparison^a^	134
No. of cases reporting mutations in overlapping target regions of TSACP and SA	104
No. of cases with additional variants found in regions unique to SA	67
No. of cases with no extra information from SA	37
No. of cases where TSACP would give a negative report	30

a Analysis was performed on a subset of the total clinical cases analyzed on the SA test in the CLIA lab.

### Statistics for off‐label use and clinical trials recommendations

The advantage of a large multigene panel is in providing relevant information pertaining to clinical trials and off‐label recommendations of drugs, particularly useful in advanced cancer care. In the SA test, off‐label drug recommendations were made only when there was an associated recruiting trial available for the relevant cancer type. Given that 40–50 drugs have been approved in the past 2 years, couple of thousands are in clinical trials and 700–800 drugs are in various stages of development, additional genetic information that could lead to the actionability of these emerging drugs is going to be relevant in the treatment of cancer 31.

Drug recommendations were analyzed in a subset of 95 cases comprising four major tissues (breast, colon, lung, and melanoma). In 60–80% of breast, melanoma, and colon cancers, off‐label recommendations were made while the number was significantly lower for lung cancer (~30%) possibly due to the presence of large number of targeted therapy options already available. Overall in >90% of all cases, the SA test augmented the reports with additional findings on variants associated with clinical trials (Fig. 5D, Table S17).

### Utility of combining IHC/MSI in the SA test

The SA test that combines NGS with tissue‐specific markers for IHC and a PCR‐based MSI test for colorectal cancers was performed on 115 samples across four tissues, breast, colon, lung (non–small cell lung cancer), and melanoma. Both presence and absence of the markers confer relevant therapeutic information as well as clinical information. The selected markers are associated with early‐line treatment involving chemotherapy or targeted therapy. Markers tested by NGS mainly provide information relevant for using targeted therapy (breast: HER2 IHC; colon: KRAS; lung: EGFR; and melanoma: BRAF). Markers tested by IHC on the other hand are predominantly associated with chemotherapy response with comparatively fewer markers for targeted therapy response (PD‐L1, HER2 among others). About 15.6% of the colon cancer cases tested positive by MSI matching what is reported in literature 32. Indeed it was observed that by combining IHC/MSI with NGS, positive reports could be sent out in 100% cases for the four tissues tested for the SOC setting. Overall, seven negative reports were issued by the SA test all for tissues other than breast, colon, lung, and melanoma.

### Utility of investigational report

The SA test provides additional clinically relevant information from the full investigational report, covered primarily by the large NGS panel. Of the 115 cases in the four tissues where a SOC report was generated, further utility was provided in 110 (96%) cases as a result of additional NGS markers covering investigational therapeutic options which increased the number for second‐ and third‐line therapy options in these cases (Table S2B).

The potential benefits of exploratory therapies are highlighted using examples listed below:

1. In a case of anaplastic thyroid cancer, a *RET‐NCOA4* translocation was detected, an event usually reported in papillary thyroid carcinoma, but uncommon in anaplastic thyroid carcinoma where drug trials targeting *RET* translocations are ongoing 33.
2. In a triple negative breast cancer case, *EGFR* amplification was detected along with a *PIK3R1* deletion. A combination of anti‐EGFR drugs and inhibitors of the PI3K‐mTOR pathway was suggested for this case 34.
3. A potential activation of the HGF‐MET axis was reported in a thymoma case, a rare cancer with no approved drugs. Interestingly, both these genes which form a receptor–ligand pair were amplified implying potential sensitivity to drugs targeting this pathway. Clinical trials with drugs targeting MET, such as cabozantinib, crizotinib, were recommended in this case 35, 36.
4. A preferential response to fulvestrant instead of aromatase inhibitors was indicated in a case of ER‐positive breast cancer. *FGFR1* amplification as well as a mutation in *ER* indicated poor response to tamoxifen 37, 38. Additionally, preclinical studies indicate that the presence of this *ER* mutation leads to ligand independent activation and is hence likely to render poor response to aromatase inhibitors 39, 40. Fulvestrant, which works by degrading the estrogen receptor, may be a viable option for this patient.

## Discussion

Making the right therapy decisions for cancer treatment is critical to ensure maximum efficacy, minimum side effects, and reduced treatment costs. Here, we describe the SA test combining the NGS and IHC/MSI technologies to strike the right balance of gene and protein markers relevant to treating early‐ and late‐stage solid tumors.

We compared the content of the SA test with other popular available tests and found an overlap of 38% with a large 315‐gene panel test which includes only NGS (http://foundationone.com/genelist1.php). The additional genes on the 315‐gene panel are either involved in germline risk prediction, hematological cancers, or have low immediate therapeutic relevance with associated drugs in preclinical development or in early phase‐I trials. It does not include IHC/MSI, therefore lacking information on early‐line treatment. The inclusion of IHC in the SA test is justified by the high number of therapeutically relevant IHC markers (>95%) reported in the SOC settings. Another popular test which includes IHCs covers only 47 genes in the NGS panel 41. First, this panel would miss a majority of the second‐ and third‐line therapy options as demonstrated by our comparison of the SA test to the 48‐gene hotspot TSACP panel. Second, the SA test integrates the results obtained from NGS and IHC tests. In this manner, the combined effect of multiple modulators for a drug is taken into account and presented. For example, response to anti‐HER2 therapies, such as lapatinib, trastuzumab, and pertuzumab, is modulated by *PIK3CA* and other downstream markers in the pathway. The SA assay and the bioinformatics pipeline have been validated extensively on a range of variants using a large number of control and clinical samples to ensure accurate and reliable results. The results were confirmed with orthogonal methods wherever possible. The SA test described here is thus an accurate and robust test that provided clinically relevant information for >95% of samples tested.

## Conflict of Interest

R.H. is the founder and CEO and V.V. is the CSO of Strand Life Sciences Pvt. Ltd. (Bangalore, India). All other authors except C.J.B. are employees of Strand Life Sciences. The authors have no financial involvement with any organization or entity with a financial interest in or financial conflict with the subject matter or materials discussed in the manuscript apart from those disclosed ahead. C.J.B. is an employee of ProPath LLC where the IHC/MSI tests are performed.

## Supporting information


**Figure S1.** The figure depicts identification of a translocation using probe designed at a known breakpoint in Exon 35 of *ROS1*. The probe hybridizes to chimeric molecules with sufficiently large overlap (>50% of the read). The fusion product depicted here has a novel translocation partner, *EPB41L2*, identified by this approach. The novel fusion product was confirmed using Sanger sequencing.

**Table S1.** (A) Samples list for validation. (B). Coverage statistics of validation samples. (C). Median average coverage genewise.
**Table S2.** (A) List of variants from clinical samples (validation). (B) List of variants from clinical samples (CLIA production lab).
**Table S3.** Clinical relevance of 152 genes.
**Table S4.** SOC markers for lung, breast, colorectal, and melanoma.
**Table S5.** IHC stains and scoring.
**Table S6.** Antibodies used for IHC in the SA test.
**Table S7.** Probability of detecting SNVs (A, B) and InDels (C) at various coverages and variant allele frequencies using an in silico downsampling experiment*.
**Table S8.** (A) Constitution of pools 1, 2, and 3 and the variants present in them.
**Table S9.** (A) Sensitivity of SNV and InDel detection in three sample pools with mutations at 5%, 5–10%, and 10% (summarized with specificity). (B). Performance of SNV detection by the SA test using QMRS FFPE control sample (HD200) with known variants.
**Table S10.** (A) Second method validation for accuracy of SNV calls.(B) Comparison of SNV calling between the SA test and TSACP.
**Table S11.** SNV/InDels specificity calculations on (A) HapMap cell line NA12878 and (B) QMRS FFPE.
**Table S12.** Copy number (amplifications and deletions) detected in control cell lines.
**Table S13.** Second method validation for accuracy of CNV calls.
**Table S14.** Limit of detection of translocations at varying tumor percentages.
**Table S15.** Second method validation for accuracy of translocations.
**Table S16.** SA test versus 48‐gene test.
**Table S17.** List of off‐label and clinical trial recommendations.
**Table S18.** Frequently mutated genes in SOC tissues.
