# Supplementary material for: StrandAdvantage test for early‐line and advanced‐stage treatment decisions in solid tumors
Source: Cancer Med. 2017 Apr 3;6(5):883–901. doi: 10.1002/cam4.1037 (PMC5430095; doi:10.1002/cam4.1037)
Supplement: Supplementary file 1 — Figure S1. The figure depicts identification of a translocation using probe designed at a known breakpoint in Exon 35 of ROS1. The probe hybridizes to chimeric molecules with sufficiently large overlap (>50% of the read). The fusion product depicted here has a novel translocation partner, EPB41L2, identified by this approach. The novel fusion product was confirmed using Sanger sequencing. [file CAM4-6-883-s001.pdf]

Supplemental Figure S1

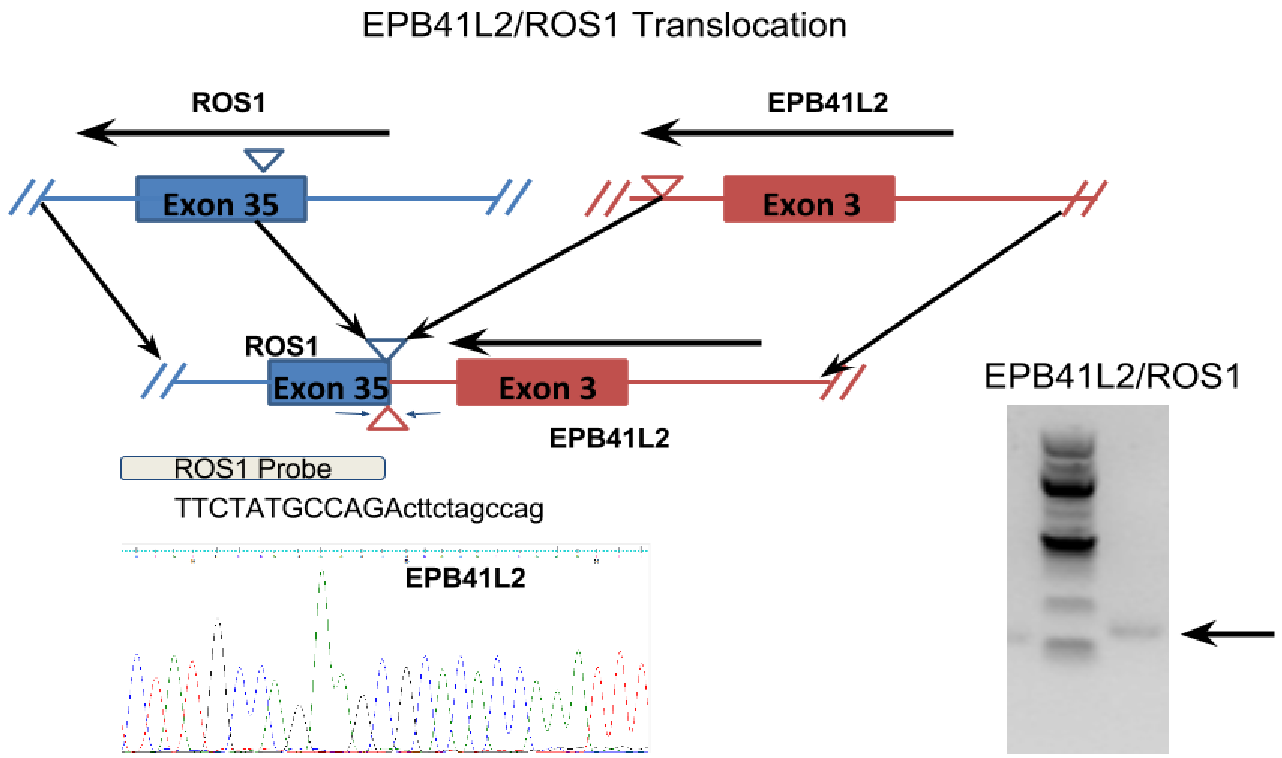

Supplemental Figure S1: The figure depicts identification of a translocation using probe designed at a known breakpoint in Exon 35 of ROS1. The probe hybridizes to chimeric molecules with sufficiently large overlap (>50% of the read). The fusion product depicted here has a novel translocation partner, EPB41L2, identified by this approach. The novel fusion product was confirmed using Sanger sequencing.
